# Supplementary material for: Seroepidemiology of human leptospirosis in the Dominican Republic: A multistage cluster survey, 2021
Source: PLoS Negl Trop Dis. 2024 Dec 23;18(12):e0012463. doi: 10.1371/journal.pntd.0012463 (PMC11735007; doi:10.1371/journal.pntd.0012463)
Supplement: S1 Table — Primary reacting serovar is defined as the serovar that registered the highest titer on microscopic agglutination test (MAT) for each seropositive study participant. Individuals that registered the same highest titer to two or more serovars are categorized as ‘mixed.’ Numbers appear to be inconsistent between Table 2 and this table given some individuals included in the ‘Mixed’ category in this table had the highest titers to two serovars from the same serogroup. For example, 13 individuals categorized as ‘Mixed’ in this table registered the highest titers to serovars Mankarso and Icterohaemorrhagiae; given these are both serogroup Icterohaemorrhagiae they are categorized by serogroup rather than ‘Mixed’ in Table 2. (DOCX) [file pntd.0012463.s001.docx]

**Table S1. Seroprevalence of *Leptospira* by primary reacting serovar, Espaillat and San Pedro de Macoris Provinces, Dominican Republic, July-Oct 2021**

| **Species** | **Serogroup** | **Primary reacting serovar** | **Strain** | **No. of positive MATs** | **Prevalence, %** | **Proportion of positive MATs, %** |
| --- | --- | --- | --- | --- | --- | --- |
| NA | NA | Mixed | NA | 42 | 2.01 | 17.6 |
| *L. interrogans* | Icterohaemorrhagiae | Mankarso | Mankarso | 40 | 1.91 | 16.8 |
| *L. interrogans* | Australis | Bratislava | Jez-Bratislava | 38 | 1.82 | 16.0 |
| *L. interrogans* | Icterohaemorrhagiae | Icterohaemorrhagiae | RGA | 38 | 1.82 | 16.0 |
| *L. interrogans* | Canicola | Canicola | Ruebush | 25 | 1.20 | 10.5 |
| *L. interrogans* | Djasiman | Djasiman | Djasiman | 19 | 0.91 | 8.0 |
| *L. santarosai* | Pyrogenes | Alexi | HS 616 | 8 | 0.38 | 3.4 |
| *L. interrogans* | Pomona | Pomona | Pomona | 5 | 0.24 | 2.1 |
| *L. borgpetersenii* | Ballum | Ballum | Mus 127 | 4 | 0.19 | 1.7 |
| *L. borgpetersenii* | Tarassovi | Tarassovi | Perepelitsin | 4 | 0.19 | 1.7 |
| *L. interrogans* | Bataviae | Bataviae | Van Tienen | 3 | 0.14 | 1.3 |
| *L. santarosai* | Mini | Georgia | LT 117 | 2 | 0.10 | 0.8 |
| *L. interrogans* | Pyrogenes | Pyrogenes | Salinem | 3 | 0.14 | 1.3 |
| *L. interrogans* | Sejroe | Wolffi | 3705 | 3 | 0.14 | 1.3 |
| *L. interrogans* | Autumnalis | Autumnalis | Akiyami A | 1 | 0.05 | 0.4 |
| *L. weilii* | Celledoni | Celledoni | Celledoni | 1 | 0.05 | 0.4 |
| *L. kirschneri* | Cynopteri | Cynopteri | 3522 C | 1 | 0.05 | 0.4 |
| *L. interrogans* | Australis | Australis | Ballico | 0 | 0.00 | 0.0 |
| *L. santarosai* | Hebdomadis | Borincana | HS 622 | 0 | 0.00 | 0.0 |
| *L. kirschneri* | Grippotyphosa | Grippotyphosa | Moskva V | 0 | 0.00 | 0.0 |
| *L. borgpetersenii* | Javanica | Javanica | Veldrat Bataviae 46 | 0 | 0.00 | 0.0 |
|  |  |  |  | **237** | **11.33** | **100** |

Primary reacting serovar is defined as the serovar that registered the highest titer on microscopic agglutination test (MAT) for each seropositive study participant. Individuals that registered the same highest titer to two or more serovars are categorized as ‘mixed.’ Numbers appear to be inconsistent between **Table 2** and this table given some individuals included in the ‘Mixed’ category in this table had the highest titers to two serovars from the same serogroup. For example, 13 individuals categorized as ‘Mixed’ in this table registered the highest titers to serovars Mankarso and Icterohaemorrhagiae; given these are both serogroup Icterohaemorrhagiae they are categorized by serogroup rather than ‘Mixed’ in **Table 2.**
