# Supplementary material for: Seroepidemiology of human leptospirosis in the Dominican Republic: A multistage cluster survey, 2021
Source: PLoS Negl Trop Dis. 2024 Dec 23;18(12):e0012463. doi: 10.1371/journal.pntd.0012463 (PMC11735007; doi:10.1371/journal.pntd.0012463)
Supplement: S2 Table — FOI, force of infection; DIC, deviance information criterion. (DOCX) [file pntd.0012463.s002.docx]

**Table S2. Priors, model estimates and DIC for serocatalytic models**

| **Model** | **Priors** | **Parameters** | **DIC** |
| --- | --- | --- | --- |
| Model 1: Catalytic  (no waning) | FOI: Uniform ~0, 0.5 | FOI = 0.0030 (0.0027 - 0.0035) | 1418 |
| Model 2a: Reverse catalytic (waning included) | FOI: Uniform ~0, 5 | FOI = 0.010 (0.007 – 0.017) | 1449 |
|  | Waning: gamma ~ (shape 28, scale 0.005) | Waning = 0.070 (0.042 – 0.12) |  |
| Model 2b: Reverse catalytic (waning included) | FOI: Uniform ~0, 5 | FOI = 0.012 (0.009 – 0.0017) | 1433 |
|  | Waning: gamma ~ (shape 28, scale 0.005) | Waning = 0.078 (0.051 – 0.120) |  |

FOI, force of infection; DIC, deviance information criterion.
