# Supplementary material for: Seroepidemiology of human leptospirosis in the Dominican Republic: A multistage cluster survey, 2021
Source: PLoS Negl Trop Dis. 2024 Dec 23;18(12):e0012463. doi: 10.1371/journal.pntd.0012463 (PMC11735007; doi:10.1371/journal.pntd.0012463)
Supplement: S3 Table — Data on national reported leptospirosis cases was from the Pan American Health Organization (PAHO) Core Indicator Dashboard from data generated by the General Epidemiology Directorate of the Ministry of Public Health and Social Assistance. (DOCX) [file pntd.0012463.s003.docx]

**Table S3. National reported leptospirosis cases, Dominican Republic 2013-2022**

| **Year** | **N** | **Female, N (%)** | **Male, N (%)** |
| --- | --- | --- | --- |
| 2013 | 139 | 26 (19) | 113 (81) |
| 2014 | 117 | 32 (27) | 85 (73) |
| 2015 | 61 | 9 (15) | 52 (85) |
| 2016 | 34 | 11 (32) | 23 (68) |
| 2017 | 36 | 8 (22) | 28 (78) |
| 2018 | 583 | 118 (20) | 465 (80) |
| 2019 | 386 | 93 (24) | 293 (76) |
| 2020 | 212 | 50 (24) | 162 (76) |
| 2021 | 305 | 83 (27) | 222 (73) |
| 2022 | 391 | 90 (23) | 301 (77) |
| **Total** | **2264** | **520 (23)** | **1774 (77)** |

Data on national reported leptospirosis cases was from the Pan American Health Organization (PAHO) Core Indicator Dashboard from data generated by the General Epidemiology Directorate of the Ministry of Public Health and Social Assistance.
