# Supplementary material for: Seroepidemiology of human leptospirosis in the Dominican Republic: A multistage cluster survey, 2021
Source: PLoS Negl Trop Dis. 2024 Dec 23;18(12):e0012463. doi: 10.1371/journal.pntd.0012463 (PMC11735007; doi:10.1371/journal.pntd.0012463)
Supplement: S4 Table — Number in dataframe = 2091, Number in model = 2089, Missing = 2, AIC = 1367.8, C-statistic = 0.721, H&L = Chi-sq(8) 1.93 (p = 0.983). N = 237 seropositive cases. San Pedro de Macoris province (Southeast study region). Espaillat province (Northwest study region). Seropositive defined as ≥ 1:100 titers using the microscopic agglutination test. (DOCX) [file pntd.0012463.s004.docx]

**Table S4. Odds ratios for testing seropositive for any *Leptospira* serogroup, Espaillat and San Pedro de Macoris Provinces, Dominican Republic, July-Oct 2021**

| **Population characteristic** | **Seronegative** | **Seropositive** | **Univariable Odds Ratio** | **Multivariable Odds Ratio** |
| --- | --- | --- | --- | --- |
|  | **N (%)** | **N (%)** | **(95% CI, p-value)** | **(95% CI, p-value))** |
| **Age** |  |  |  |  |
| 5-19 | 384 (97.2) | 11 (2.8) | Ref | Ref |
| 20-34 | 480 (90.4) | 51 (9.6) | **3.71 (1.98-7.59, p<0.001)** | **3.87 (2.05-7.98, p<0.001)** |
| 35-49 | 398 (86.3) | 63 (13.7) | **5.53 (2.99-11.22, p<0.001)** | **5.57 (2.97-11.41, p<0.001)** |
| 50-64 | 341 (86.8) | 52 (13.2) | **5.32 (2.84-10.91, p<0.001)** | **5.07 (2.67-10.48, p<0.001)** |
| 65+ | 251 (80.7) | 60 (19.3) | **8.34 (4.48-17.04, p<0.001)** | **6.77 (3.58-13.96, p<0.001)** |
| **Gender** |  |  |  |  |
| Female | 1236 (92.3) | 103 (7.7) | Ref | Ref |
| Male | 605 (82.2) | 131 (17.8) | **2.60 (1.97-3.43, p<0.001)** | **2.41 (1.79-3.25, p<0.001)** |
| Other | 13 (81.2) | 3 (18.8) | 2.77 (0.63-8.76, p=0.116) | 2.89 (0.63-9.63, p=0.114) |
| **Study region** |  |  |  |  |
| San Pedro de Macorís | 1170 (91.4) | 110 (8.6) | Ref | Ref |
| Espaillat | 684 (84.3) | 127 (15.7) | **1.97 (1.51-2.60, p<0.001)** | **1.84 (1.31-2.58, p<0.001)** |
| **Setting** |  |  |  |  |
| Urban | 1071 (90.8) | 109 (9.2) | Ref | Ref |
| Rural | 783 (85.9) | 128 (14.1) | **1.61 (1.22-2.11, p=0.001)** | 1.19 (0.88-1.61, p=0.263) |
| **Occupation** |  |  |  |  |
| Non-professional | 1742 (89.6) | 202 (10.4) | Ref | Ref |
| Farmer | 48 (64.9) | 26 (35.1) | **4.67 (2.80-7.63, p<0.001)** | 1.63 (0.94-2.80, p=0.078) |
| Professional | 64 (87.7) | 9 (12.3) | 1.21 (0.56-2.35, p=0.596) | 1.05 (0.47-2.08, p=0.906) |
| **Contact with rats** |  |  |  |  |
| No | 1561 (88.8) | 196 (11.2) | Ref | Ref |
| Yes | 291 (87.7) | 41 (12.3) | 1.12 (0.77-1.59, p=0.529) | **1.64 (1.06-2.52, p=0.024)** |

Number in dataframe = 2091, Number in model = 2089, Missing = 2, AIC = 1367.8, C-statistic = 0.721, H&L = Chi-sq(8) 1.93 (p=0.983). N = 237 seropositive cases. San Pedro de Macoris province (Southeast study region). Espaillat province (Northwest study region). Seropositive defined as ≥ 1:100 titers using the microscopic agglutination test.
