# Supplementary material for: Seroepidemiology of human leptospirosis in the Dominican Republic: A multistage cluster survey, 2021
Source: PLoS Negl Trop Dis. 2024 Dec 23;18(12):e0012463. doi: 10.1371/journal.pntd.0012463 (PMC11735007; doi:10.1371/journal.pntd.0012463)
Supplement: S5 Table — Number in dataframe = 2091, Number in model = 2089, Missing = 2, AIC = 701.2, C-statistic = 0.751, H&L = Chi-sq(8) 5.53 (p = 0.699). N = 91 seropositive cases. San Pedro de Macoris province (Southeast study region). Espaillat province (Northwest study region). Seropositive defined as ≥ 1:100 titers using the microscopic agglutination test. (DOCX) [file pntd.0012463.s005.docx]

**Table S5. Odds ratios for testing seropositive for *Leptospira interrogans* serogroup Icterohaemorrhagiae, Espaillat and San Pedro de Macoris Provinces, Dominican Republic, July-Oct 2021**

| **Population characteristic** | **Seronegative** | **Seropositive** | **Univariable Odds Ratio** | **Multivariable Odds Ratio** |
| --- | --- | --- | --- | --- |
|  | **N (%)** | **N (%)** | **(95% CI, p-value)** | **(95% CI, p-value))** |
| **Age** |  |  |  |  |
| 5 to 19 | 390 (98.7) | 5 (1.3) | Ref | Ref |
| 20-34 | 513 (96.6) | 18 (3.4) | **2.74 (1.08-8.35, p=0.048)** | **2.91 (1.14-8.96, p=0.038)** |
| 35-49 | 440 (95.4) | 21 (4.6) | **3.72 (1.50-11.23, p=0.009)** | **3.77 (1.50-11.49, p=0.009)** |
| 50-64 | 373 (94.9) | 20 (5.1) | **4.18 (1.67-12.67, p=0.005)** | **3.93 (1.55-12.02, p=0.008)** |
| 65+ | 284 (91.3) | 27 (8.7) | **7.42 (3.07-22.08, p<0.001)** | **5.83 (2.37-17.59, p<0.001)** |
| **Gender** |  |  |  |  |
| Female | 1302 (97.2) | 37 (2.8) | Ref | Ref |
| Male | 683 (92.8) | 53 (7.2) | **2.73 (1.78-4.22, p<0.001)** | **2.69 (1.71-4.26, p<0.001)** |
| Other | 15 (93.8) | 1 (6.2) | 2.35 (0.13-12.06, p=0.415) | 2.37 (0.13-13.17, p=0.422) |
| **Study region** |  |  |  |  |
| San Pedro de Macorís | 1247 (97.4) | 33 (2.6) | Ref | Ref |
| Espaillat | 753 (92.8) | 58 (7.2) | **2.91 (1.89-4.55, p<0.001)** | **2.82 (1.65-4.99, p<0.001)** |
| **Setting** |  |  |  |  |
| Urban | 1146 (97.1) | 34 (2.9) | Ref | Ref |
| Rural | 854 (93.7) | 57 (6.3) | **2.25 (1.47-3.50, p<0.001)** | **1.62 (1.02-2.61, p=0.042)** |
| **Occupation** |  |  |  |  |
| Non-professional | 1863 (95.8) | 81 (4.2) | Ref | Ref |
| Farmer | 66 (89.2) | 8 (10.8) | **2.79 (1.20-5.68, p=0.009)** | 0.74 (0.31-1.62, p=0.482) |
| Professional | 71 (97.3) | 2 (2.7) | 0.65 (0.11-2.11, p=0.550) | 0.54 (0.09-1.85, p=0.412) |
| **Contact with rats** |  |  |  |  |
| No | 1680 (95.6) | 77 (4.4) | Ref | Ref |
| Yes | 318 (95.8) | 14 (4.2) | 0.96 (0.52-1.67, p=0.892) | 1.73 (0.83-3.51, p=0.133) |

Number in dataframe = 2091, Number in model = 2089, Missing = 2, AIC = 701.2, C-statistic = 0.751, H&L = Chi-sq(8) 5.53 (p=0.699). N = 91 seropositive cases. San Pedro de Macoris province (Southeast study region). Espaillat province (Northwest study region). Seropositive defined as ≥ 1:100 titers using the microscopic agglutination test.
