# Supplementary material for: Seroepidemiology of human leptospirosis in the Dominican Republic: A multistage cluster survey, 2021
Source: PLoS Negl Trop Dis. 2024 Dec 23;18(12):e0012463. doi: 10.1371/journal.pntd.0012463 (PMC11735007; doi:10.1371/journal.pntd.0012463)
Supplement: S6 Table — Number in dataframe = 2091, Number in model = 2089, Missing = 2, AIC = 364.5, C-statistic = 0.746, H&L = Chi-sq(8) 3.04 (p = 0.932). N = 38 seropositive cases. San Pedro de Macoris province (Southeast study region). Espaillat province (Northwest study region). Seropositive defined as ≥ 1:100 titers using the microscopic agglutination test. (DOCX) [file pntd.0012463.s006.docx]

**Table S6. Odds ratios for testing seropositive for *Leptospira interrogans* serogroup Australis, Espaillat and San Pedro de Macoris Provinces, Dominican Republic, July-Oct 2021**

| **Population characteristic** | **Seronegative** | **Seropositive** | **Univariable Odds Ratio** | **Multivariable Odds Ratio** |
| --- | --- | --- | --- | --- |
|  | **N (%)** | **N (%)** | **(95% CI, p-value)** | **(95% CI, p-value))** |
| **Age** |  |  |  |  |
| 5 to 19 | 393 (99.5) | 2 (0.5) | Ref | Ref |
| 20-34 | 524 (98.7) | 7 (1.3) | 2.62 (0.63-17.68, p=0.230) | 2.02 (0.47-13.75, p=0.390) |
| 35-49 | 448 (97.2) | 13 (2.8) | **5.70 (1.56-36.61, p=0.022)** | 4.23 (1.11-27.66, p=0.064) |
| 50-64 | 385 (98.0) | 8 (2.0) | 4.08 (1.02-27.17, p=0.076) | 3.06 (0.73-20.76, p=0.166) |
| 65+ | 303 (97.4) | 8 (2.6) | **5.19 (1.29-34.54, p=0.038)** | 3.08 (0.72-21.12, p=0.170) |
| **Gender** |  |  |  |  |
| Female | 1320 (98.6) | 19 (1.4) | Ref | Ref |
| Male | 717 (97.4) | 19 (2.6) | 1.84 (0.96-3.52, p=0.063) | 1.14 (0.51-2.41, p=0.743) |
| Other | 16 (100.0) |  |  |  |
| **Study region** |  |  |  |  |
| San Pedro de Macorís | 1263 (98.7) | 17 (1.3) | Ref | Ref |
| Espaillat | 790 (97.4) | 21 (2.6) | **1.97 (1.04-3.81, p=0.039)** | 1.54 (0.65-3.84, p=0.335) |
| **Setting** |  |  |  |  |
| Urban | 1169 (99.1) | 11 (0.9) | Ref | Ref |
| Rural | 884 (97.0) | 27 (3.0) | **3.25 (1.65-6.86, p=0.001)** | 2.10 (1.00-4.70, p=0.058) |
| **Occupation** |  |  |  |  |
| Non-professional | 1918 (98.7) | 26 (1.3) | Ref | Ref |
| Farmer | 65 (87.8) | 9 (12.2) | **10.21 (4.37-21.94, p<0.001)** | **5.55 (2.03-14.98, p=0.001)** |
| Professional | 70 (95.9) | 3 (4.1) | 3.16 (0.74-9.26, p=0.064) | 2.44 (0.56-7.54, p=0.165) |
| **Contact with rats** |  |  |  |  |
| No | 1728 (98.3) | 29 (1.7) | Ref | Ref |
| Yes | 323 (97.3) | 9 (2.7) | 1.66 (0.73-3.40, p=0.189) | 2.30 (0.84-6.23, p=0.098) |

Number in dataframe = 2091, Number in model = 2089, Missing = 2, AIC = 364.5, C-statistic = 0.746, H&L = Chi-sq(8) 3.04 (p=0.932). N = 38 seropositive cases. San Pedro de Macoris province (Southeast study region). Espaillat province (Northwest study region). Seropositive defined as ≥ 1:100 titers using the microscopic agglutination test.
