# Supplementary material for: Seroepidemiology of human leptospirosis in the Dominican Republic: A multistage cluster survey, 2021
Source: PLoS Negl Trop Dis. 2024 Dec 23;18(12):e0012463. doi: 10.1371/journal.pntd.0012463 (PMC11735007; doi:10.1371/journal.pntd.0012463)
Supplement: S7 Table — Number in dataframe = 2091, Number in model = 2089, Missing = 2, AIC = 264, C-statistic = 0.781, H&L = Chi-sq(8) 3.01 (p = 0.934). N = 25 seropositive cases. Reference category for age group 20–34 vs 5–19 for prior models given absence of seropositive cases in the youngest age category. San Pedro de Macoris province (Southeast study region). Espaillat province (Northwest study region). Seropositive defined as ≥ 1:100 titers using the microscopic agglutination test. NC, not calculated. (DOCX) [file pntd.0012463.s007.docx]

**Table S7. Odds ratios for testing seropositive for *Leptospira interrogans* serogroup Canicola, Espaillat and San Pedro de Macoris Provinces, Dominican Republic, July-Oct 2021**

| **Population characteristic** | **Seronegative** | **Seropositive** | **Univariable Odds Ratio** | **Multivariable Odds Ratio** |
| --- | --- | --- | --- | --- |
|  | **N (%)** | **N (%)** | **(95% CI, p-value)** | **(95% CI, p-value))** |
| **Age** |  |  |  |  |
| 5 to 19 | 395 (100.0) | 0 (0.0) | NC | NC |
| 20-34 | 526 (99.1) | 5 (0.9) | - | - |
| 35-49 | 450 (97.6) | 11 (2.4) | 2.57 (0.93-8.21, p=0.082) | 2.69 (0.96-8.74, p=0.072) |
| 50-64 | 389 (99.0) | 4 (1.0) | 1.08 (0.27-4.11, p=0.907) | 0.99 (0.24-3.86, p=0.985) |
| 65+ | 306 (98.4) | 5 (1.6) | 1.72 (0.47-6.23, p=0.395) | 1.68 (0.45-6.29, p=0.432) |
| **Gender** |  |  |  |  |
| Female | 1325 (99.0) | 14 (1.0) | - | - |
| Male | 727 (98.8) | 9 (1.2) | 1.17 (0.49-2.68, p=0.712) | 1.44 (0.59-3.34, p=0.401) |
| Other | 14 (87.5) | 2 (12.5) | **13.52 (2.01-54.82, p=0.001)** | **21.37 (2.93-102.50, p<0.001)** |
| **Study region** |  |  |  |  |
| San Pedro de Macorís | 1269 (99.1) | 11 (0.9) | - | - |
| Espaillat | 797 (98.3) | 14 (1.7) | 2.03 (0.92-4.59, p=0.081) | **3.24 (1.23-9.37, p=0.021)** |
| **Setting** |  |  |  |  |
| Urban | 1165 (98.7) | 15 (1.3) | - | - |
| Rural | 901 (98.9) | 10 (1.1) | 0.86 (0.37-1.91, p=0.718) | 0.57 (0.23-1.35, p=0.204) |
| **Occupation** |  |  |  |  |
| Non-professional | 1921 (98.8) | 23 (1.2) | - | - |
| Farmer | 74 (100.0) | 0 (0.0) | NC | NC |
| Professional | 71 (97.3) | 2 (2.7) | 2.35 (0.37-8.16, p=0.252) | 1.81 (0.28-6.63, p=0.439) |
| **Contact with rats** |  |  |  |  |
| No | 1737 (98.9) | 20 (1.1) | - | - |
| Yes | 327 (98.5) | 5 (1.5) | 1.33 (0.44-3.31, p=0.573) | 2.90 (0.80-9.85, p=0.089) |

Number in dataframe = 2091, Number in model = 2089, Missing = 2, AIC = 264, C-statistic = 0.781, H&L = Chi-sq(8) 3.01 (p=0.934). N = 25 seropositive cases. Reference category for age group 20-34 vs 5-19 for prior models given absence of seropositive cases in the youngest age category. San Pedro de Macoris province (Southeast study region). Espaillat province (Northwest study region). Seropositive defined as ≥ 1:100 titers using the microscopic agglutination test. NC, not calculated.
