# Supplementary material for: Seroepidemiology of human leptospirosis in the Dominican Republic: A multistage cluster survey, 2021
Source: PLoS Negl Trop Dis. 2024 Dec 23;18(12):e0012463. doi: 10.1371/journal.pntd.0012463 (PMC11735007; doi:10.1371/journal.pntd.0012463)
Supplement: S8 Table — Generalized variance inflation factor (GVIF) and degrees of freedom (DF) for each covariate included in the regression models. The adjusted GVIF accounts for the degrees of freedom of each covariate to simplify interpretation across covariates with different degrees of freedom. An adjusted GVIF value close to 1 indicates multicollinearity is not substantially inflating the variance of the estimated regression coefficients for that covariate. (DOCX) [file pntd.0012463.s008.docx]

**Table S8. Multicollinearity of model covariates**

| **Covariate** | **GVIF** | **DF** | **Adjusted GVIF** |
| --- | --- | --- | --- |
| Age category | 1.1 | 4 | 1.0 |
| Gender | 1.1 | 2 | 1.0 |
| Study region | 1.5 | 1 | 1.2 |
| Setting | 1.1 | 1 | 1.1 |
| Occupation | 1.2 | 2 | 1.0 |
| Contact with rats | 1.4 | 1 | 1.2 |

Generalized variance inflation factor (GVIF) and degrees of freedom (DF) for each covariate included in the regression models. The adjusted GVIF accounts for the degrees of freedom of each covariate to simplify interpretation across covariates with different degrees of freedom. An adjusted GVIF value close to 1 indicates multicollinearity is not substantially inflating the variance of the estimated regression coefficients for that covariate.
