# Supplementary figures and images for: Seroepidemiology of human leptospirosis in the Dominican Republic: A multistage cluster survey, 2021
Source: PLoS Negl Trop Dis. 2024 Dec 23;18(12):e0012463. doi: 10.1371/journal.pntd.0012463 (PMC11735007; doi:10.1371/journal.pntd.0012463)

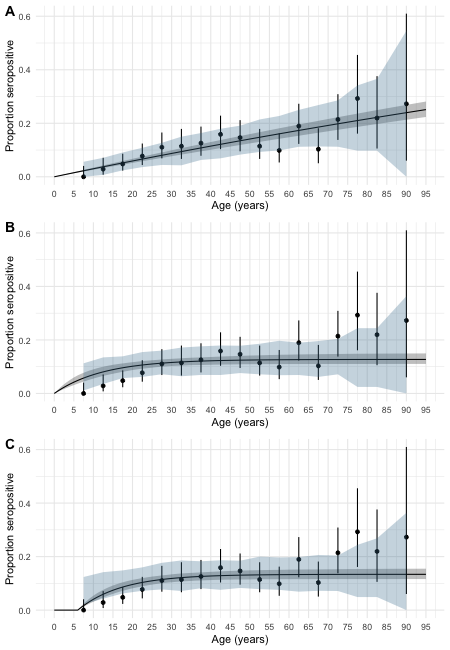

Supplement: S1 Fig — (A) Catalytic model. (B) Reverse catalytic model. (C) Reverse catalytic model with no transmission in the six years prior to the serosurvey. (PNG) [file pntd.0012463.s009.png]

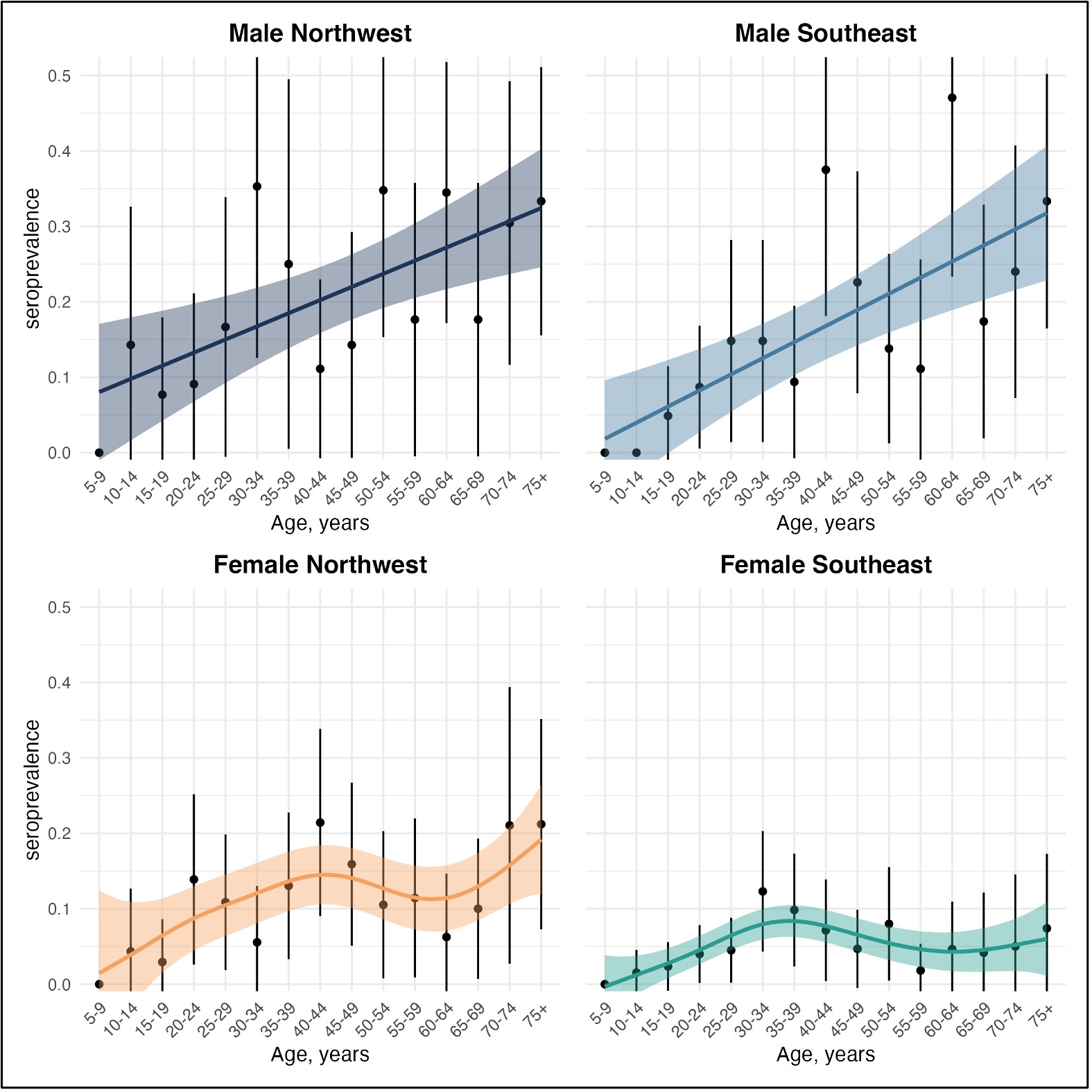

Supplement: S2 Fig — Individual plots show seroprevalence point estimates (black dots) and 95% confidence intervals (black vertical lines) by five-year age group. A smoothed line is fitted to the data using generalized additive models with a cubic spline and weighted for number of observations. Colored ribbons represent the 95% CI. (JPG) [file pntd.0012463.s010.jpg]

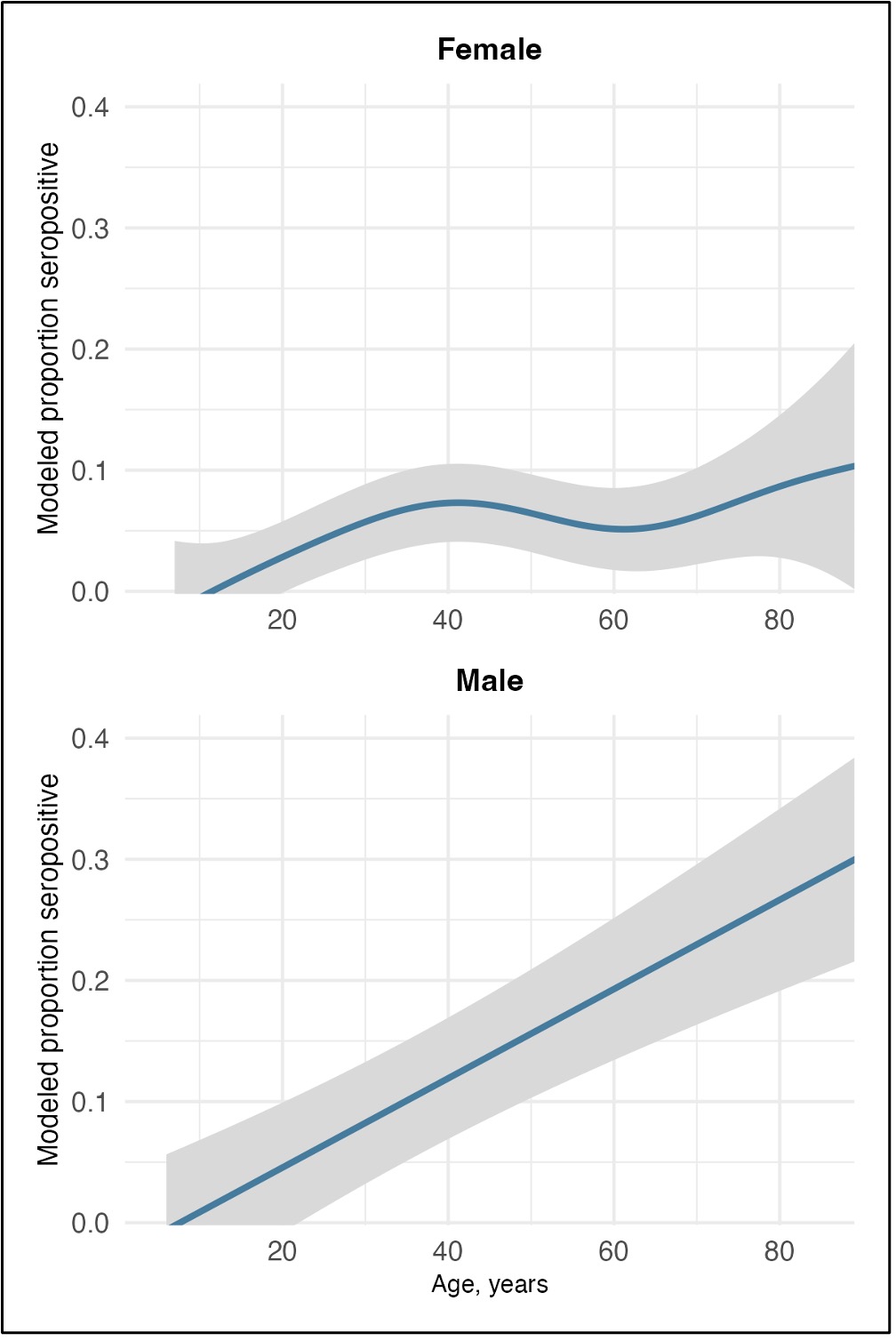

Supplement: S3 Fig — Generalized Additive Models indicate the proportion of individuals with Leptospira antibodies (titers ≥1:100), stratified by gender. The models included smooth terms for age to capture non-linear age effects and adjusted for study region, residing in a barrio (settlement), work environment, and rural vs. urban residence. Ribbons represent the 95% CI. (JPG) [file pntd.0012463.s011.jpg]
