# Supplementary material for: Seroepidemiology of human leptospirosis in the Dominican Republic: A multistage cluster survey, 2021
Source: PLoS Negl Trop Dis. 2024 Dec 23;18(12):e0012463. doi: 10.1371/journal.pntd.0012463 (PMC11735007; doi:10.1371/journal.pntd.0012463)
Supplement: Attachment — Submitted filename: Response to reviewer comments.docx [file pntd.0012463.s012.docx]

**Response to reviewer comments**

**Comment: #1** The manuscript describes a seroepidemiological study of leptospirosis in the Dominican Republic. Due to the lack of laboratory diagnosis and awareness, leptospirosis is underdiagnosed, and surveillance data underestimates its true impact. This study is an important contribution, demonstrating the role of seroepidemiological investigations in populations with limited knowledge of leptospirosis. It is a cost-effective approach to obtain valuable epidemiological information, generate hypotheses, and inform improvements in diagnosis and surveillance systems.

**Response:** The authors thank the reviewer for their time and thoughtful comments.

The manuscript reinforces the fact that leptospirosis is underestimated and provides a good case study and methodological details that can be useful for others. However, some important limitations should be considered and addressed:

**Comment #2:** MAT is the standard methodology for the determination of Leptospira-specific antibodies and positivity means past exposure. Authors hint at MAT's limitation in identifying infecting serogroups but still analyzed the data, interpreted results, and made conclusions as it reflects circulating serogroups. For the serogroup sub-analysis, I appreciated they conducted a sensitivity analysis but the report should elaborate more on the limitation and make clear that they are simply reporting serogroups with the highest reacting titers. They cannot discuss results or make conclusions about circulating serogroups. Any comparison with other studies should reflect this as well.

**Response:** Thank you for highlighting this point, with which we agree. We have made the following edits that hopefully address this concern.

- Added to discussion/limitations (L349): “Irrespective, given well established limitations of MAT for definitely identifying the infecting serogroup, and given we were reporting putative serogroups based on the highest reacting titer, our serogroup-specific findings should be considered as suggestive rather than definitive.”
- Concluding paragraph (L364) edited from “Analyzing serogroups individually identified distinct transmission patterns” to “Analyzing serogroups individually suggested distinct transmission patterns”.

**Comment #3:** I appreciate the effort to estimate FOI and inferences about the expected number of infections, including examining different models and assumptions. It is recognized that many infections are missed; this exercise demonstrates that. Authors inferred 33,000 infections per year, much higher than the reported counts; however, authors are comparing expected infections with the number of people who were ill enough to seek care, then tested, and reported. I don’t think those are comparable. The spectrum of leptospirosis illness is broad and a fraction of people may have subclinical disease, self-limiting mild illness, etc. and therefore would never make it into the reportable system. Others are ill but not cannot access care. Others seek care but they are not tested. The paper should be clear about the different comparisons they want to make, their purpose, and impact.

**Response:** We fully agree with the reviewer that there are a range of reasons for under-ascertainment of leptospirosis. We did not, however, intend to imply that infections equate to clinically significant disease (or clinically significant disease that is correctly diagnosed and reported) and tried to be careful with our terminology to ensure readers understood that we were reporting differences in clinically reported cases to estimated infections. This is, in our opinion, a fairly standard approach —used across a range of pathogens — when using serological data to estimate under-ascertainment.

Overall, we certainly tried to ensure that what we were comparing was clear. For example:

- L36: ‘and a marked discrepancy between reported cases and serologically estimated infections’
- L276: ‘marked difference between national reported cases and infections.’
- L280: ‘we used serological data to estimate the annual attack rate and in turn the difference between nationally reported leptospirosis cases and serologically estimated infections.’
- L287: ‘the ratio of reported cases to estimated infections ranged from about 1:28 to 1:140’

However, there are two sentences that would certainly benefit from clarification, and these have been edited as follows:

- L282: ‘Our findings suggest marked under ascertainment with 100-fold or greater difference in reported versus estimated cases’ changed to ‘Our findings suggest marked under ascertainment with 100-fold or greater difference in reported clinical cases versus estimated infections.’
- L366: ‘and a substantial discrepancy between reported and likely cases’ changed to ‘and a substantial discrepancy between reported cases and likely infections.’

Additionally, given the importance of this point, we added the following text to the discussion (L:291): The marked discrepancy between reported cases and estimated infections likely reflects factors such as subclinical or mild infections that do not prompt medical care, limited access to healthcare or leptospirosis testing, and gaps in clinician awareness of leptospirosis symptoms, which may hinder diagnosis.

**Comment #4:** Authors conducted various analyses of sub-groups, and found highest rates among the older population. There was no attempt to explain this finding by providing some ideas for behaviors or exposure that could explain the findings. For example, is it that people at those ages may switch to spending more time at home and there is an increase in domestic exposures?

**Response:** We certainly found the age distribution unexpected. In fact, the rationale for performing multiple subgroup analyses was to better understand this finding. Unfortunately, despite these analyses — that included stratifying by region, sex, urban vs rural, and both sex and region — we were not able to generate a satisfactory explanation. We certainly considered a range of possibilities such as very high transmission around the 1950s that could result in an unusual pattern of immune imprinting in the older population (based upon the concept that repeated antigenic exposures result in more durable immune responses). Certainly, the hypothesis that an increase in domestic exposure is possible. However, the increase in leptospirosis infection risk (and seroprevalence) observed near-universally during early adulthood is widely considered to be due to occupational exposures, so this hypothesis would run counter to the general consensus. Similarly, if domestic exposure was driving infection risks, we would anticipate increased seroprevalence among younger children, under most circumstances. Overall, the hypotheses we considered appeared quite speculative and could not be meaningfully supported by our data. Therefore we did make the decision not to include these in the manuscript.

**Comment #5:** MAT titers are only displayed in a figure and not mentioned in the results and discussion. MAT titers can provide additional information about the transmission dynamics. It seems like most are low titers, 1:100. Results can highlight the profiles of those with high titers.

**Response:** Thank you for highlighting this oversight. We have added text describing the distribution of titers, both overall and stratified by putative serogroup (L225).
